# Recurrent Biatrial Myxomas in Carney Complex with a Spinal Melanotic Schwannoma: Advocacy for a Rigorous Multidisciplinary Follow-Up

**DOI:** 10.1155/2023/7896180

**Published:** 2023-12-11

**Authors:** Daniel Grandmougin, Teresa Moussu, Maxime Hubert, Benjamin Perin, Arthur Huber, Maria Christina Delolme, Juan-Pablo Maureira

**Affiliations:** ^1^Department of Cardiac Surgery, CHU Nancy-Brabois, France; ^2^Cardiology Practice, Hagondange, France; ^3^Department of Anesthesiology, CHU Nancy-Brabois, France

## Abstract

A 31-year-old female patient with a previous history of Carney complex and surgical resection for cardiac myxoma and bilateral adrenalectomy at 18 years old and 10 and 11 years old, respectively, was referred to our department with a diagnosis of recurrent biatrial myxomas incidentally discovered on echography. A magnetic resonance imaging (MRI) confirmed the diagnosis of a tumor protruding into the left ventricle, and the patient underwent a surgical resection of a large left atrial mass and a right-sided atrial small tumor. Diagnosis of bilateral atrial myxomas was confirmed by histologic studies. Postoperative outcome was uneventful, and the patient was discharged at the 7th postoperative day. Few months later, she reported trivial clinical symptoms suspecting a cervical radiculopathy. MRI confirmed the presence of a compressive cervical spinal cord tumoral mass at the C2-3 level leading to perform a surgical exeresis of the tumor. Histology showed a spinal melanotic schwannoma. This case highlights the risk of unexpected ubiquitary tumor locations and the importance of a rigorous transversal multidisciplinary follow-up to prevent severe complications in patients with Carney complex.

## 1. Introduction

Carney complex (CNC) is a rare autosomal syndrome resulting from either a dominant inheritance (70%) or a sporadic occurrence (30%) as a de novo genetic defect [1], described by Carney et al. in 1985 [2]. Although the prevalence remains unclear, largest genotyped cohorts of patients show a marked female predominance (63% versus 37%) [3, 4].

CNC is characterized by a multiple neoplasia syndrome featuring myxomas of the heart, breast nipples, bone, skin, and mucosae and endocrine and nonendocrine, cutaneous, and neural tumors, as well as a variety of abnormal skin pigmentation and a predisposition to cancers such as thyroid, ovaries, pancreas, and liver [1, 3]. If clinical characteristics of CNC are likely present at birth, nonetheless the median age of diagnosis is known to be around 20 years of age. However, sporadic mutations usually produce mild symptoms later in life [1, 5]. Therefore, a close and regular follow-up is crucial to detect new symptoms and avoid life-threatening complications and recurrence of tumoral process [6].

We report the case of a 31-year-old woman, known to have a CNC and referred to our department with a diagnosis of paucisymptomatic recurrent large left atrial myxoma. Characteristics of CNC, surgical management, and recommendations for follow-up will be discussed from a literature review.

## 2. Case Presentation

A 31-year-old woman was admitted to our department for a recurrent left atrial tumor whose echographic features were relevant with a myxoma.

**Figure 1:**
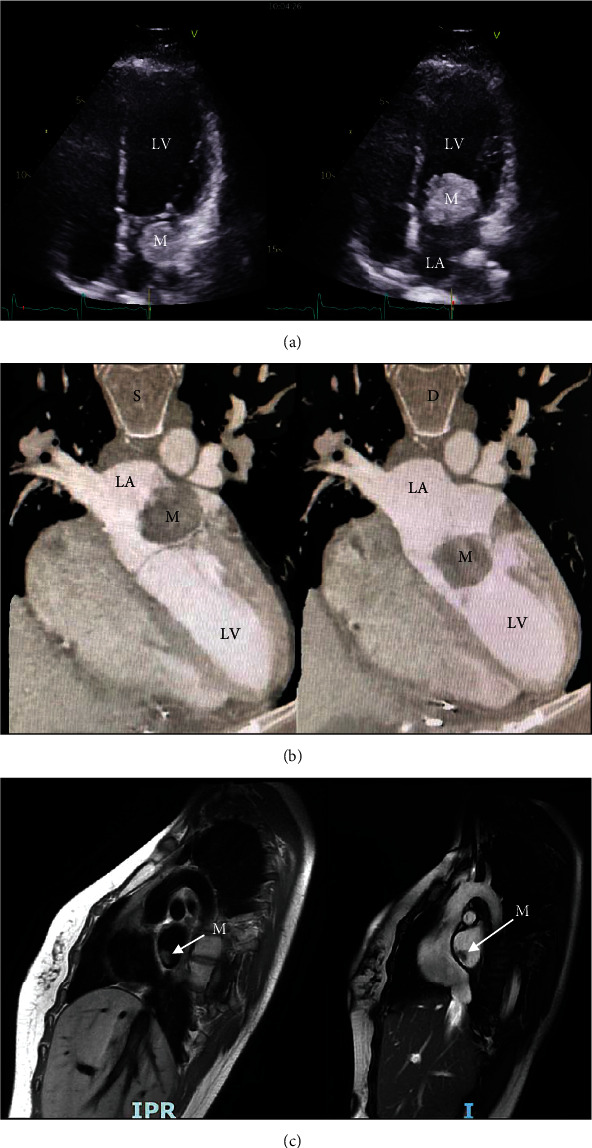
(a) Four-chamber echographic view shows a mobile left atrial mass (M) protruding into the left ventricle (LV) whose ultrasonic characteristics are consistent with a myxoma (LA: left atrium). (b) MRI performed in 2021 shows the mass (M) protruding through the mitral valve from the left atrium (LA) during systole (S) into the left ventricle (LV) during diastole (D). (c) MRI (sagittal T2- and T1-weighted images) performed in 2007 shows the first occurrence of a 17 × 14 mm left atrial mass (M). Video link on YouTube: https://www.youtube.com/watch?v=cPPDHg0cYZc.

The atrial mass was incidentally discovered by echography (Figure 1(a) (video link on YouTube: https://www.youtube.com/watch?v=cPPDHg0cYZc)). MRI (Figure 1(b)) revealed a large and mobile left atrial mass (32 × 35 mm) protruding through the mitral valve into the left ventricle in diastole and consistent with a myxoma as the patient reported a recent and insidious shortness of breath, with neither chest pain nor fever and weight loss. She was known to have a CNC, nevertheless with an irregular follow-up. Her medical history included a bilateral adrenalectomy at 10 and 11 years old, respectively, for a primary pigmented nodular adrenocortical disease with hypercortisolism and Cushing's syndrome. At the age of 18, she had developed a left atrial myxoma (17 × 14 mm) attached to the interatrial septum (Figure 1(c)) which was surgically removed. The septum had been reconstructed with a pericardial patch. Since then, she had remained asymptomatic and unfortunately neglected her cardiac follow-up. However, at the age of 27, diagnosis of CNC was definitely validated as a genetic analysis of PRKAR1A gene showed presence of the mutation c.172delGAGAAGGGTAAA. ECG showed a sinus rhythm. Plasmatic levels of chromogranin A and growth hormone were <30 ng/ml and 6.3 ng/l, respectively. Because of a high risk of intracardiac obstruction due to mitral protrusion, surgery was rapidly scheduled.

### 2.1. Operative Procedure

**Figure 2:**
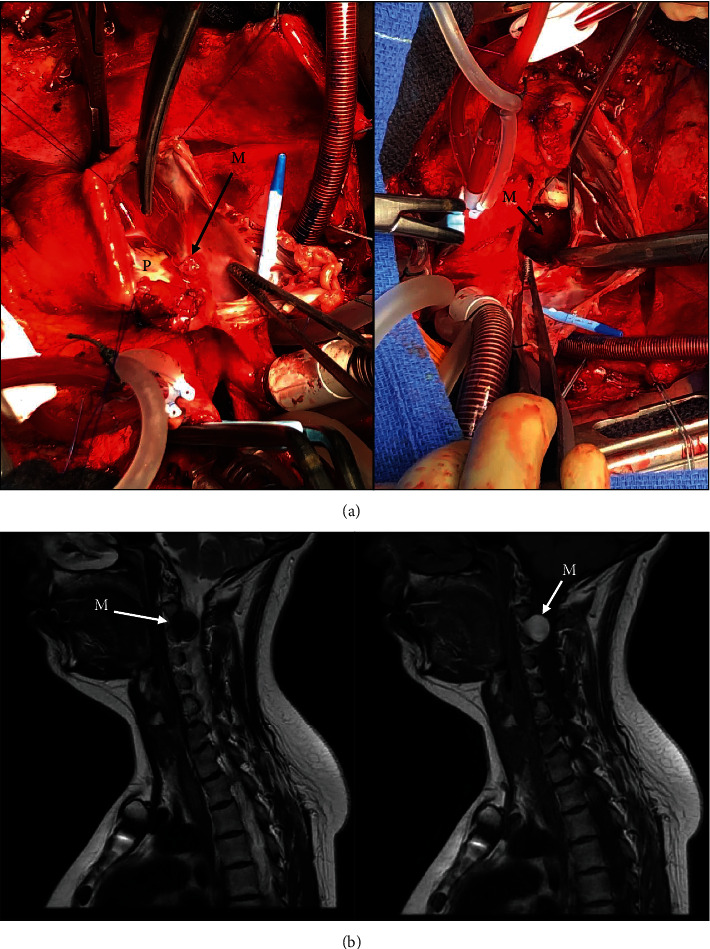
(a) Operative views. Left part: after right atriotomy and before septotomy: a small 7 × 6 mm mass (M) enshrined below the insertion of the previous pericardial patch (P) used to reconstruct the interatrial septum 13 years earlier. Right part: after septotomy, a gelatinous and mobile mass (M) is easily visualized into the left atrium. (b) Cervical MRI performed 11 months after the resection of the myxoma depicts a 20 × 18 mm cervical intradural spherical mass (M) at the C2-3 level with signs of spinal cord compression. Left part: T2-weighted sagittal image. Right part: T1-weighted sagittal image.

After a median resternotomy and liberation of pericardial adherences, aortic and venous cannulation, the latter through the superior vena cava and the femoral vein percutaneously, were performed for normothermic cardiopulmonary bypass. The left myxoma was approached through a superior biatrial septotomy showing a mobile and gelatinous mass found attached to the posterior mitral (P2) annulus by a small and thin stalk. After resection, examination of the right atrial chamber showed a small (7 × 6 mm) gelatinous mass enshrined below the insertion of the previous pericardial patch and easily resected (Figure 2(a)). The postoperative pathological analysis confirmed a myxoma of both masses with no malignant characteristics.

### 2.2. Follow-Up

Postoperative outcome was uneventful, and the patient was discharged in good conditions with awareness to respect a close medical monitoring through regular outpatient visits. Eleven months later, while reporting dysesthesia with a moderate cutaneous hypoesthesia around the neck, the patient underwent a cervical MRI which disclosed a 20 × 18 mm cervical intradural spherical mass at the C2-3 level with signs of spinal cord compression and without any osteolytic component (Figure 2(b)). The tumor was successfully resected, and pathological examination revealed a melanotic schwannoma.

## 3. Discussion

CNC is a rare multiple neoplasia and lentiginosis syndrome with an autosomal dominant inheritance (70%) or can occur sporadically (30%), initially described by Carney et al. in 1985 [2] and definitely named “Carney complex” in 1986 [3]. CNC is characterized by multiple endocrine and nonendocrine tumors involving the heart with specific risks of life-threatening complications, pigmented skin lesions, neural tumors, predisposition to multiple cancers, and endocrine overactivity such as an overproduction of cortisol resulting from a specific tumor in CNC, named primary pigmented nodular adrenocortical disease (PPNAD), and leading to develop a Cushing's syndrome. The peak of diagnosis of PPNAD is usually during the second and third decades of life [1, 5]. However, histologic evidence of PPNAD has been found in almost every individual with CNC who underwent autopsy, thus showing that a number of patients had asymptomatic PPNAD. It is interesting to note that our patient was diagnosed at the first decade and required a bilateral adrenalectomy at 10 and 11 years old, respectively.

Therefore, patients with CNC necessitate a rigorous endocrine follow-up including plasma level monitoring of chromogranin A and growth hormone, since excess of this latter has been associated in 60% of patients with myxomas or recurrence of myxomas [5, 6]. In our case, on admission, only plasmatic level of growth hormone was elevated.

In 70% of patients, onset of CNC is related to mutations of the PRKAR1A gene located on the long arm of chromosome 17. This percentage increases to 80% for patients with Cushing's syndrome due to primary PPNAD. The PRKARI1A gene codes for the regulatory subunit type I alpha (R1A) of the protein kinase A enzyme. Therefore, PRKAR1A is a major protein involved in AMPc signaling pathway known to influence the endocrine tumorigenesis and hence might mediate tumor suppression [4, 5].

Additionally, other candidate genes or loci may be screened including the PRKACA (chromosome 19) and PRKACB (chromosome 1) [1, 2, 4, 5].

Cardiac myxomas are histologically benign tumors representing 45% of primary cardiac tumors in adult. Left atrial location is the most common (75%) followed by right atrial involvement (18%). Biatrial location is a rare situation (2.5 to 4%). Up to 7% of cardiac myxomas are associated with CNC. In a group of 319 CNC patients, Pitsava et al. [3] showed that 136 of them (42.6%) developed at least one cardiac myxoma, independently of a PRKAR1A mutation, located in the left atrium (64%), the right atrium (13.46%), the left ventricle (9.6%), and the right ventricle (6.7%). Cardiac myxomas are the leading cause of death in patients with CNC because they may be responsible of intracardiac obstruction, strokes due to fragmentation of the tumor with emboli [7], myocardial infarction, and sudden death, and hence, surgery is mandatory to avoid a dismal prognosis. Incidence of recurrence after surgical resection is low (1 to 3%). On the other hand, in CNC patients, the incidence of recurrence is high and ranges between 10 and 44% [3, 5, 6, 8]. If gender does not influence the initial occurrence of myxomas, on the other hand, it obviously plays a significant role in recurrence of myxomas since, in the study published by Pitsava et al. [3], 73.3% of recurrence were females. Therefore, women are 2.5 times more likely to experience a recurrence compared to men. In addition, presence of skin or mucosal lentigines, cutaneous or mucosal myxomas, breast myxomatosis, thyroid lesions, pituitary adenomas, and psammomatous melanotic schwannomas might predict the presence of a myxoma [3, 4]. In our case, the occurrence of a delayed spinal melanotic schwannoma, eleven months after a recurrence of cardiac myxomas, highlights the importance of a rigorous follow-up of CNC patients, at least once a year, with a multidisciplinary approach including a psychological management, a complete education, and information given to the patients and the family to avoid a loss of follow-up with dire consequences mainly related to a high risk of developing cardiac myxomas or multiple tumors and then improve prognosis as well as life expectancy [5–8]. Therefore, screening for cardiac myxoma by echography should start early during the first 6 months and continued for the rest of the life once a year or biannually if a cardiac myxoma has been already excised. Furthermore, our experience showed that awareness of both cardiologists and cardiac surgeons to CNC is a key point to detect patients. Actually, recommendations for clinical and imaging follow-up mainly focus on the risk of life-threatening complications such as sudden death related to both a high incidence and recurrence of cardiac myxomas. In our case, due to a diagnosis of an insidious melanotic schwannoma with poor clinical complaints, if regular cardiac echography remains crucial, several discussions with internists led us to recommend to perform, once a year, a whole-body CT scan and/or MRI to detect tumors independently of their locations and characteristics since they may have a rapid tumor growth nevertheless with the potential for deleterious consequences.

Decision to perform either a CT scan and/or MRI depends on several factors such as the age and sex of the patient, anatomic locations and characteristics of the tumor to be assessed, the intended surgical procedure, and the overall medical history of the patient including pregnancy desire.

If CT scan offers an interesting spatial resolution and covers a large anatomic surface useful to detect tumor extension and metastasis, nevertheless repeat CT scans remain problematic because of cumulative radiation dose levels. In contrast, MRI allows an optimal tissular characterization without any radiation particularly interesting for tumors.

Additionally, PET-CT has a better sensitivity and specificity to detect small tumors, nodules, or metastasis with lower radiation levels.

More recently, developments and investigations of whole-body low-field MRI [9, 10] opened a promising path to a radiation-free alternative to both whole-body CT scan and PET-CT.

Therefore, in order to organize a specific and reactive follow-up within a multidisciplinary collaborative team, the role of a referent internist is essential to select optimal imaging exams considering patient characteristics.

In our case, the erratic follow-up, observed from the second to the third decade, interfered with a rigorous strategy for therapy and follow-up and might have had a pejorative cardiac issue. This key point underlines the importance of patient education to improve life expectancy which should be started in childhood with an adapted and regular psychological support.

## 4. Conclusions

This case emphasized the importance of early diagnosis of CNC to improve life expectancy. Rigorous guidelines for a transversal multidisciplinary follow-up are crucial since failure in follow-up may have dismal consequences.

## Data Availability

Additional data used to support the findings of this study are available from the corresponding author upon request.

## Abbreviations

CNC: Carney complex
PPNAD: Primary pigmented nodular adrenocortical disease
MRI: Magnetic resonance imaging
ECG: Electrocardiogram.
